# The effect of micronutrient supplementation on women’s sexual desire: a systematic review and meta-analysis study

**DOI:** 10.1590/1806-9282.20260669

**Published:** 2026-10-23

**Authors:** Nurçin Küçük Kent, Sevda Karakaş, Sevda Uzun

**Affiliations:** 1 Gümüşhane University, Department of Emergency Aid and Disaster Management – Gümüşhane, Türkiye.; 2 Gümüşhane University, Faculty of Health Sciences, Department of Nursing – Gümüşhane, Türkiye.; 3 Kırşehir Ahi Evran University, Faculty of Health Sciences, Department of Nursing – Kırşehir, Türkiye.

**Keywords:** Micronutrients, Women, Sexual desire, Meta-analysis

## Abstract

**OBJECTIVE:**

The aim of this study was to determine the effectiveness of micronutrient supplementation on female sexual desire levels.

**METHODS:**

For this systematic review and meta-analysis, studies were identified by searching the PubMed, Web of Science, ScienceDirect, Google Scholar, and Higher Education Council (YÖK) Thesis Center databases without any year restrictions. After screening, eight studies were included in the analysis. Data were synthesized using meta-analysis and narrative methods.

**RESULTS:**

In this systematic review and meta-analysis, micronutrient supplementation was associated with a statistically significant effect on women’s sexual desire (standardized mean difference: -0.597, 95%CI -1.126 to -0.068, Z=-2.211, p=0.027); however, substantial heterogeneity was observed (I^2^ =87.2%) indicating considerable variability across studies.

**CONCLUSION:**

This systematic review suggests a potential role for micronutrients, but the evidence is weak and highly inconsistent. High-quality, low-heterogeneity research is urgently needed.

## INTRODUCTION

Micronutrients (vitamins and minerals) contribute to maintaining the physiological homeostasis of sexual desire and sexual function in women and to preventing the development of potential sexual dysfunction by playing a role in the regulation of gonadal functions and in the steroidogenesis process of sex-specific hormones^1,2^. Micronutrients, particularly zinc, vitamin D, B vitamins, and antioxidant vitamins, play a supportive role in maintaining women’s sexual health and improving potential sexual dysfunction through their effects on hormonal balance, neurotransmitter activity, and vascular function^3,4^. It is known that micronutrient deficiency is a significant risk factor for sexual aversion in women. Sexual aversion, one of the most common issues among sexual dysfunctions in women, ranks among the most prevalent disorders, with a prevalence rate of 50.7%^5,6^. Studies in the literature report that micronutrient supplementation (zinc, vitamin D, and B vitamins) provides significant benefits in maintaining women’s sexual health and improving sexual desire by supporting hormone synthesis, neurotransmitter balance, and overall metabolic functions^6,7,8^. Zinc may contribute to the regulation of sexual desire by playing a role in the synthesis of gonadal hormones, including estrogen and progesterone. The presence of vitamin D receptors in the reproductive system and brain tissue suggests that this vitamin may influence libido through its effects on sexual function and mood. It has been suggested that B vitamins may indirectly increase sexual desire and arousal by supporting neurotransmitter synthesis through serotonergic and dopaminergic pathways^8,9^. This study was conducted to investigate the effectiveness of micronutrient supplementation on women’s sexual desire levels.

## METHODS

This systematic review and meta-analysis was prepared in accordance with the PRISMA checklist (Preferred Reporting Items for Systematic Reviews and Meta-Analyses Protocols)^10^. To minimize the risk of potential bias in this systematic review and meta-analysis, the literature search, article selection, and data extraction processes were conducted independently by three researchers. These steps were subsequently reviewed by the three researchers. The quality assessment of the studies included in the systematic review and meta-analysis was also performed by the researchers.

### Inclusion criteria

In this study, studies were screened according to PICOS:

P: Population: Women
I: Intervention: Micronutrient use
C: Comparison: No use of micronutrients
C: Outcomes: Sexual desire
S: Study design: Experimental studies published in Turkish and English.

### Exclusion criteria

Studies in the form of letters to the editor, case–control studies, and systematic and traditional reviews were excluded from the scope of this research.

### Search strategy

The search was conducted through ScienceDirect, Web of Science, YÖK TEZ, and Google Scholar up to March 2026, using the keywords “sexual desire and women,” “micronutrients and women and sexual desire,” or “micronutrients and women,” in accordance with Medical Subject Headings (MeSH) criteria. A search was conducted on PubMed using appropriate MESH terms (“Sexual Desire”[MESH] OR “Sexual Dysfunction, Physiological”[MESH]) AND (“Female”[MESH]) AND (“Micronutrients”[Mesh] OR “Vitamins”[Mesh] OR “Minerals”[Mesh] OR “Dietary Supplements”[Mesh]). Since the number of studies on the effects of micronutrient supplementation on women’s levels of sexual desire is limited, no year restrictions were applied, and all years were included in the search.

### Selection of studies

The search initially yielded 146 records. After removing duplicate studies, 131 records were reviewed based on their titles and abstracts for selection. As a result of this review, 48 studies were selected for full-text analysis. Subsequently, the 31 articles for which full texts were obtained were reviewed according to inclusion and exclusion criteria, and 8 studies reporting results on the effects of micronutrient supplementation on women’s sexual desire levels were included in the analysis^11,12,13,14,15,16,17,18^. Explanations regarding the article selection process are provided in Figure 1. This study is a systematic review and meta-analysis and was conducted based on studies published in the literature.

**Figure 1:**
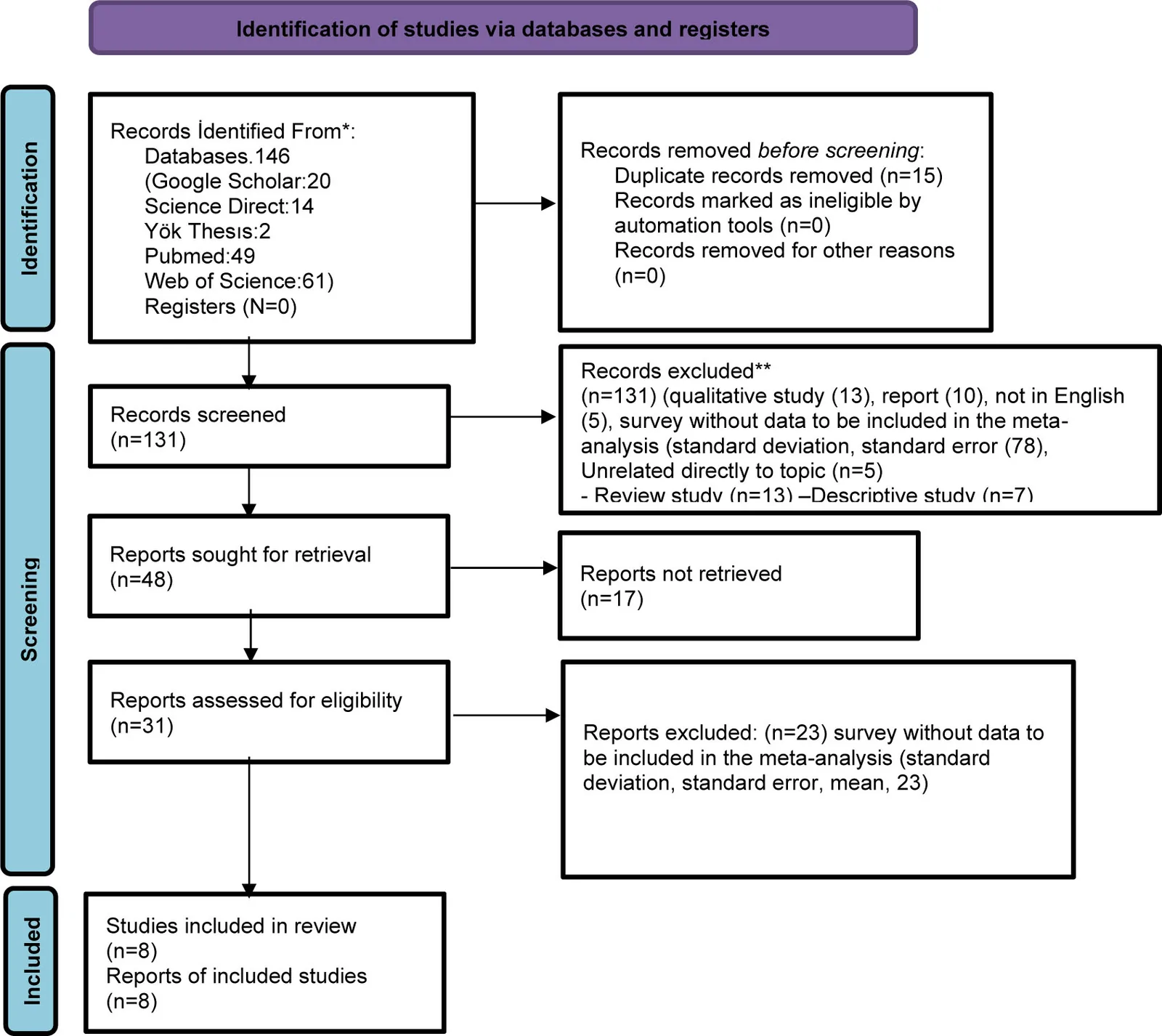
PRISMA. *Databases searched: Google Scholar, ScienceDirect, YÖK Thesis, PubMed, and Web of Science. **Reasons for exclusion: qualitative studies, reports, studies not published in English, surveys without sufficient data for meta-analysis, studies not directly related to the topic, review studies, and descriptive studies.

### Assessment of the methodological quality of the studies

The quality assessment of the studies included in this systematic review and meta-analysis was conducted by the Joanna Briggs Institute using quality assessment tools tailored to the study design. The assessment tools used in this study were selected based on the designs of the studies included in the systematic review and meta-analysis. In our study, a 13-item assessment tool was used for randomized controlled studies (The Joanna Briggs Institute Critical Appraisal Tools for Use in IBI Systematic Reviews, 2021), and a 9-item assessment tool was used for quasi-experimental studies. The methodological quality of the included studies was categorized based on the percentage of “Yes” responses to the 13 JBI criteria: ≥70% was considered low risk of bias (high quality), 50–69% as moderate risk of bias (moderate quality), and <50% as high risk of bias (low quality). No studies were found to have a high risk of bias. In this study, the evaluation results for each study are shown in Table 1 as “*Quality score*”^19^.

**Table 1:** Characteristics and main findings of the studies included in the systematic review and meta-analysis.

Author/year	Study design	Measurement tools	Sample size/characteristics	Duration of intervention	Food used in the intervention	Main results	Quality scores
Izadi et al. ^11^	Randomized controlled trial	Female Sexual Function Index (FSFI)	Experimental group: 25 Control group: 25	8 weeks	Saffron	An increase in sexual desire was observed in the experimental group.	Yes: 13/13
Valizadeh et al.^13^	Randomized controlled trial	Female Sexual Function Index (FSFI)	Experimental group: 30 Control group: 29	8 weeks	Probiotic	An increase in sexual desire was observed in the experimental group.	Yes: 13/13
Mazaheri Nia et al.^10^	Randomized controlled trial	Female Sexual Function Index (FSFI)	Experimental group: 30 Control group: 29	8 weeks	Zinc	An increase in sexual desire was observed in the experimental group.	Yes: 9/13 No: 2/13 Uncertain: 1/13 Not applicable: 1/13
Ghamari et al.^12^	Randomized controlled trial	Female Sexual Function Index (FSFI)	Experimental group: 31 Control group: 31	8 weeks	Ginseng	An increase in sexual desire was observed in the experimental group.	Yes: 13/13
Jahanian Sadatmahalleh et al.^14^	Randomized controlled trial	Female Sexual Function Index (FSFI)	Experimental group: 35 Control group: 35	8 weeks	Vitamin D	An increase in sexual desire was observed in the experimental group.	Yes: 13/13
Sarebani et al.^17^	Randomized controlled trial	Female Sexual Function Index (FSFI)	Experimental group: 33 Control group: 32	8 weeks	Vitamin D	An increase in sexual desire was observed in the experimental group.	Yes: 9/13 No: 2/13 Uncertain: 1/13 Not applicable: 1/13
Rafati et al.^15^	Randomized controlled trial	Female Sexual Function Index (FSFI)	Experimental group: 20 Control group: 20	4 weeks	Vitamin D	An increase in sexual desire was observed in the experimental group.	Yes: 13/13
Ito et al.^16^	Randomized controlled trial	Female Sexual Function Index (FSFI)	Experimental group: 34 Control group: 43	8 weeks	Arginmax	An increase in sexual desire was observed in the experimental group.	Yes: 13/13

### Data synthesis

For the statistical calculations of this study, CMA Ver. 2 was used. The heterogeneity between the studies was evaluated with the Chi-squared statistic and Higgins I^2^ tests, and an I^2^ of more than 50% was considered to indicate significant heterogeneity^19^. Studies with I^2^≤50% were evaluated using a fixed effects model; however, studies with I^2^>50% were evaluated using a random effects model. A random effects model was used in this study. Statistical significance was considered at p<0.05 for all tests^19,20^.

In addition, the Tau-squared statistic was used to complete the assessment of variance and heterogeneity between studies. The standardized mean difference (SMD) with 95%CI was used to assess the effect size associated with the same outcome with different measurement tools. Furthermore, forest plots were prepared to visualize the SMD with 95%CI. These D values were then averaged to calculate the overall effect size. The D value was converted to a Z value to assess statistical significance. Funnel plots were used to examine and visualize publication bias. Publication bias was detected by an asymmetry in the funnel plot. The Begg test was also performed to assess publication bias^19,20^.

Sensitivity analysis was performed by excluding one study at a time and evaluating whether there was a significant change in the results; there was no significant change in the results^19,20^.

## RESULTS

The selection of studies for this systematic review and meta-analysis according to the PRISMA flow diagram is presented in Figure 1.

The characteristics and main findings of the studies are presented in Table 1; it was determined that the studies met more than half of the items in the evidence quality assessment tool (Table 1).

### Meta-analysis results on the effectiveness of micronutrient supplementation regarding women’s sexual desire level

In this study, the presence of publication bias was determined using two methods: (a) a funnel plot and (b) the Begg and Mazumdar correlation test. In the funnel plot—one of the key methods for detecting publication bias—we observe that the studies in this dataset are clustered at the top of the funnel and exhibit a symmetrical distribution. This indicates the absence of publication bias (Figure 2).

**Figure 2:**
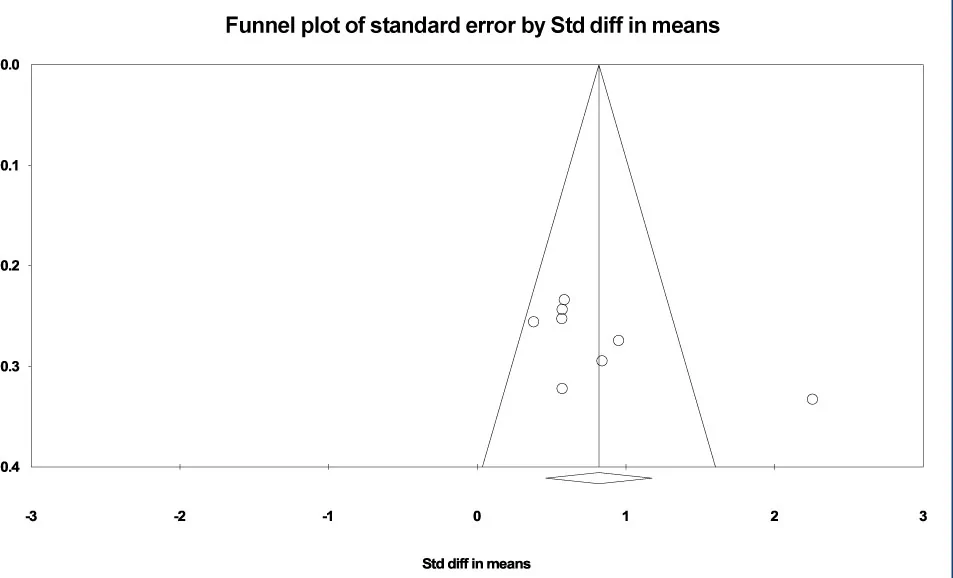
Funnel plot of studies reporting results on the effectiveness of micronutrient supplementation on women’s sexual desire levels.

Publication bias among the studies included in this dataset was determined using the Begg and Mazumdar rank correlation method. According to the rank correlation method, the two-sided p-value is 0.22. This result indicates that the publication bias is not statistically significant.

Figure 3 presents a forest plot of the eight studies examining the effect of micronutrient supplementation on women’s sexual desire levels, including effect sizes, standard errors, variances, and lower and upper limits. Based on the findings of these studies, a meta-analysis concluded that micronutrient supplementation is effective in improving women’s sexual desire levels (SMD: -0.597, 95%CI -1.126 to -0.068; Z=-2.211, p=0.027, I^2^=87.2%) (Figure 3).

**Figure 3:**
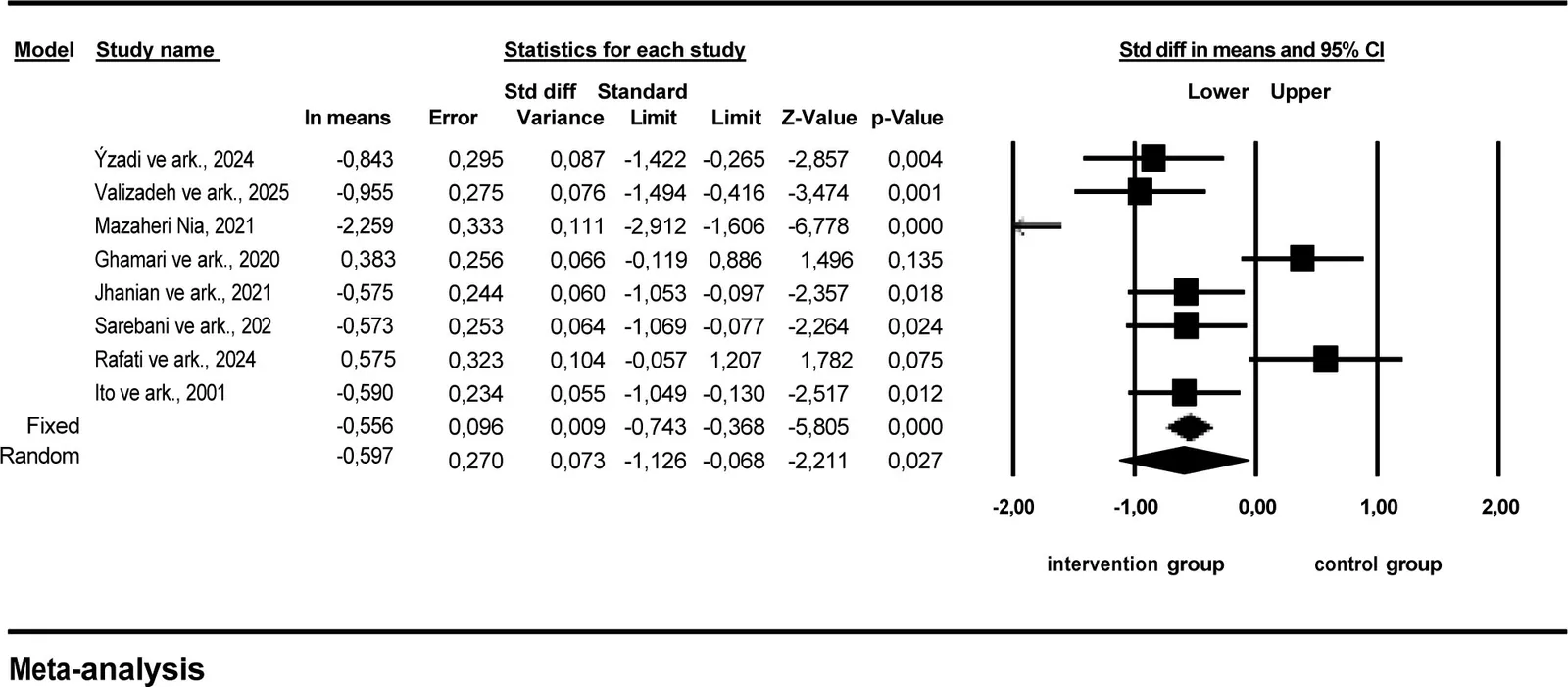
Forest plot showing the effect of micronutrient supplementation on women’s sexual desire levels in the intervention and control groups.

## DISCUSSION

This study was conducted to systematically review randomized controlled trials evaluating the effectiveness of micronutrient supplementation on female sexual desire and to perform a meta-analysis of the existing evidence. The findings of this systematic review and meta-analysis suggest that micronutrient supplementation may improve female sexual desire; however, the certainty of the available evidence is limited. Similar to our study, Canat et al. found in their study involving 50 women with low sexual desire that micronutrient supplementation (vitamin D) was effective in increasing women’s sexual desire levels^3^. The effects of vitamins A, C, E, D, and B on women’s sexual health have been examined in the literature; it has been reported that vitamins A, C, E, D, and B complex vitamins have beneficial effects on women’s sexual function^8-12^. In particular, study results have reported that vitamin D and niacin (B3) have effects that increase women’s sexual desire^9-11^. The pooled findings suggest that micronutrient supplementation may have a beneficial effect on women’s sexual desire; however, this finding should be interpreted with caution due to the substantial heterogeneity and variability in study designs. Female sexual desire disorder is a multifactorial condition, and therefore a single standardized treatment approach is not appropriate. Existing literature suggests that micronutrient supplementation may positively influence female sexual function through potential mechanisms such as supporting hormonal balance, reducing oxidative stress, regulating neurotransmitter synthesis, and improving vascular function^11,21,22^. The management of female sexual desire disorder requires a multidisciplinary approach, typically beginning with individualized psychotherapy and, when appropriate, proceeding to pharmacological treatments and, in rare cases, surgical interventions. Within this framework, existing literature suggests that micronutrient supplementation may positively influence female sexual function through potential mechanisms such as supporting hormonal balance, reducing oxidative stress, regulating neurotransmitter synthesis, and improving vascular function; therefore, adequate vitamin and mineral intake may have a supportive role in maintaining sexual function^12-16^. In contrast to our study, Ghanbari et al. reported in their 2023 study involving 337 women that vitamin supplementation (including vitamins A, C, E, and B) did not result in a significant improvement in female sexual function, that the results were inconsistent, and that the overall level of evidence was insufficient^23^. It is believed that the discrepancy in results is related to differences in intervention procedures and inclusion criteria. Therefore, although this systematic review suggests a potential role for micronutrient supplementation in improving female sexual desire, the available evidence remains weak and highly heterogeneous. Future well-designed randomized controlled trials with larger sample sizes, standardized intervention protocols, and validated outcome measures are urgently needed to provide more robust evidence.

## CONCLUSION AND RECOMMENDATIONS

This systematic review and meta-analysis suggest that micronutrient supplementation may improve women’s sexual desire; however, the current evidence is weak and highly inconsistent, and the certainty of the available evidence is limited. Additionally, it is recommended that randomized controlled trials be conducted with larger sample sizes to evaluate the effectiveness of micronutrient supplementation on women in terms of various variables. Future studies should also consider evaluating additional psychosocial and biological variables that may influence women’s sexual desire in order to provide a more comprehensive understanding of the effects of micronutrient supplementation.

## LIMITATIONS OF THE STUDIES

Some of the studies included in the systematic review and meta-analysis had small sample sizes and were conducted without blinding. These factors may reduce the strength of the evidence provided by the studies.

## Data Availability

The datasets generated and/or analyzed during the current study are available from the corresponding author upon reasonable request.
